# Striking the balance: exploring the influence of exercise quantity and intensity on quality-of-life outcomes

**DOI:** 10.1016/j.clinsp.2025.100782

**Published:** 2025-09-29

**Authors:** Ronaldo Alves da Cunha, Claudio Andre Barbosa de Lira, Rodrigo Luiz Vancini, Katja Weiss, Beat Knechtle, Marilia Santos Andrade

**Affiliations:** a Postgraduate Program in Translation Medicine, Universidade Federal de São Paulo, São Paulo, SP, Brazil; b Human and Exercise Physiology Division, Faculdade de Educação Física e Dança da Universidade Federal de Goiás, Goiânia, GO, Brazil; c Center for Physical Education and Sports, Universidade Federal do Espírito Santo, Vitória, ES, Brazil; d Institute of Primary Care, University of Zurich, Zurich, Switzerland; e Medbase St. Gallen Am Vadianplatz, St. Gallen, Switzerland; f Department of Physiology, Universidade Federal de São Paulo, São Paulo, SP, Brazil

**Keywords:** Exercises, Physical health, Mental health, SF-36, Well-being

## Abstract

**Background and aims:**

The World Health Organization (WHO) recommends physical activity for a better quality of life (QoL). However, it is unknown if a lesser or an additional physical activity than the WHO recommendation impacts the QoL. The aim of the study was to verify whether people who engage in physical activity above or below the levels recommended by the WHO show differences in quality of life.

**Methods:**

The International PA Questionnaire (IPAQ) was used to determine the PA characteristics, and the 36-Item Short-Form Health Survey (SF-36) to QoL (SF-36 scores range from 0–100, and a higher score indicates better QoL). A total of 180 men (44.9 ± 14.5 years) were divided into: Group 1 (< 150 min moderate or < 75 min vigorous PA per week); Group 2 (150–600 min of moderate PA or 75–300 min of vigorous PA per week); and Group 3 (> 600 min moderate or > 300 min vigorous PA per week). Results: Group 1 presented significantly lower scores than Group 2 for the overall physical domain (mean difference = 15.5, 95% IC = 22.1 to 8.9, p < 0.001) and overall mental domain (mean difference = 19.1, 95% IC = 27.5 to 10.6, p < 0.001). Group 2 presented similar results to group 3 for the overall physical domain (mean difference = -0.5, 95% IC = 7.1 to -8.2, p = 1.00) and overall mental domain (mean difference = 3.1, 95% IC = 12.9 to -6.6, p =1.00).

**Conclusion:**

Individuals who comply with the amount of weekly physical activity recommended by the WHO have better QoL (physical and mental) than those who do less physical activity than recommended; however, those who do much more physical activity than the recommended amount do not present additional benefits for QoL.

## Highlights

• More physical activity than recommended by the WHO does not generate additional gains in quality of life (physical or mental).
• Quality of life (physical and mental) is better among those who perform the WHO physical activity recommendation than among those who perform less physical activity.
• There is no linear relationship between physical activity and quality of life.

## Introduction

Physical activity plays an important role in the primary prevention and treatment of several different psychiatric, neurological, cardiovascular, pulmonary, musculoskeletal, and metabolic diseases^1^^,^^2^. Indeed, over the last three decades, a myriad of studies have emerged on the effects of physical activity, and nowadays there is considerable knowledge concerning the possible mechanisms that can justify the benefits of physical activity^1^^,^^3^.

Accordingly, in the last decade, the general recommendations for physical activity proposed at least 150 min/week of moderate-intensity aerobic exercise, 75 min/week of vigorous-intensity aerobic exercise, or a combination of both^4^. However, in 2020, given the well-known multiple health-related benefits of physical activity, the World Health Organization (WHO) released an update to the guidelines on physical activity for the entire population from all age groups^5^^,^^6^, increasing the minimum recommended level. Nowadays, it is recommended that all adults should undertake 150–300 min/week of moderate-intensity or 75–150 min/week of vigorous-intensity physical activity, or an equivalent combination^5^, suggesting that a greater volume of physical activity per week may produce higher health benefits and better Quality of Life (QoL). In addition to volume, the intensity of physical activity also seems to be important. An overview of systematic reviews concluded that vigorous exercise has been shown to be associated with greater improvements in depression and anxiety than moderate exercise^7^.

Conversely, very high-intensity or large-volume exercises can present negative health effects. For example, excessive physical activity can lead to overtraining syndrome, characterized by fatigue, decreased performance, and increased injury risk (Bereda, 20,22^8^.) Considering mental health, excessive exercise has also been linked to depression and anxiety, lowering the QoL and the individual's performance^9^^,^^10^.

Accordingly, it is the consensus in the literature that a minimum of 150–300 min of moderate activity or 75–150 min of vigorous activity should be performed for health benefits and QoL; however, it is unknown if a higher volume than this recommendation is capable of producing greater QoL.

Therefore, the aim of the present study was to compare the health quality among subjects who exercise less, equally, or much more than the WHO recommendation. The hypothesis of the study is that individuals who exercise according to the WHO minimum exercise recommendation would have a better QoL than those who exercise less, but that those who exercise much more than the recommendation do not present higher QoL.

## Methods

### Study design

This was a cross-sectional study conducted between November 2019 and March 2020. The study was approved by the Medical Ethics Committee of the Federal University of Sao Paulo (Approval Number 4.354.386). After a detailed explanation of the questionnaire that should be answered, the participants signed an informed consent form.

### Participants

This research employed a convenience sampling strategy to recruit participants. Male individuals older than 18-years were invited to participate in the study through flyer cards distributed around the university, in parks, and via social media channels. The criteria for inclusion were meticulously defined to ensure a homogeneous participant profile conducive to the study’s objectives. The inclusion criteria were to be free from pain or musculoskeletal injuries at the time of the study, have no history of severe injuries, and not suffer from chronic or mental health conditions. The exclusion criteria were to complete the entire questionnaire. Ultimately, the study successfully enrolled a total of 180 men who met these criteria.

This study was conducted in accordance with the Strengthening the Reporting of Observational Studies in Epidemiology (STROBE) guidelines.

### Evaluation

Participants’ body height and body mass were measured using a calibrated wall stadiometer and a balance (Filizola®, São Paulo, Brazil), respectively. Body Mass Index (BMI) was calculated from the person’s weight in kilograms divided by the square of their height in meters.

All participants completed a paper-based questionnaire to investigate personal characteristics and current medical conditions, followed by an evaluation of overall physical and mental health status and physical activity level. Participants were divided into three groups: Group 1, those who performed less than the WHO recommendation (< 150 min/week of moderate exercise and < 75 min/week of vigorous exercise); Group 2, those who performed the WHO recommendation or up to twofold more (150–600 min/week of moderate exercise or 75–300 min/week of vigorous exercise); and Group 3, those who overreached the recommendation (> 600 min/week of moderate exercise or > 300 min/week of vigorous exercise).

### Physical and mental health status

Self-reported physical, mental, and emotional health status was evaluated with the Portuguese-validated version of the Short Form Health Survey (SF-36), which is a useful clinical tool to evaluate health-related QoL^11^^,^^12^. The SF-36 was self-administered and consists of 36 items grouped into eight subdomains: physical functioning, physical role functioning, bodily pain, general health perception, vitality, social functioning, emotional role functioning, and mental health. All scales are scored from 0 to 100, with a higher score indicating better health status. These eight subdomains can be further aggregated into two main domains, indicating overall physical- and mental-health-related QoL^13^. For analysis purposes, the mean values of the four physical and four mental domains were calculated, and these values reflect the overall physical and mental health status.

### Physical activity level

The International Physical Activity Questionnaire (IPAQ) was used to assess the weekly total minutes expended with moderate and vigorous exercise, and also the physical activity level of the volunteers. This instrument was validated for Brazilian Portuguese^14^. According to the answers provided by the participants, the total weekly minutes expended with moderate and vigorous exercises were quantified. In addition, the level of physical activity was classified into five categories: very active, active, irregularly active A, irregularly active B, and not active. As the very active category encompasses all participants who reached or exceeded the weekly physical activity (in minutes and intensity) recommended by the WHO, this classification was not used in the present study.

### Data analysis

Descriptive data were presented as the mean and standard deviation. All data presented a normal distribution according to the Shapiro-Wilk test, and homogenous variances were confirmed using Levene’s test. The sample size was calculated using a significant effect size of 0.30, which is considered a medium effect^15^, a significance level of 0.05, and a power of 0.80. The G*Power program (Version 3.1.9.7; Heinrich-Heine-Universität Düsseldorf, Düsseldorf, Germany) was used to analyze the test power level. According to the calculations, a total sample size of 111 participants was required. The general characteristics and the SF-36 answers were compared among the three physical activity groups using one-way analysis of variance (ANOVA). The ANOVA was supplemented with the Bonferroni post hoc test when the significance threshold was met. Partial eta squared is presented as a measure of effect size. The significance level was set at 0.05. All statistical analyses were performed using SPSS Version 26.0 (IBM, Inc., Chicago, IL).

## Results

There was no statistically significant difference in the age and height of the participants among Groups 1, 2 and 3. In contrast, Group 1 presented higher values for body mass and BMI than Groups 2 and 3 (Table 1). As expected, the mean values for weekly minutes spent with moderate and vigorous physical activity were significantly different among the three groups. In Group 2 the weekly vigorous physical activity was close to the WHO recommendation, but in Group 1, it was much lower than the recommendation; conversely, in Group 3, it was much higher than the recommendation (Table 1).

**Table 1:** General characteristics of the participants.

	Group 1 (*n* = 95)	Group 2 (*n* = 41)	Group 3 (*n* = 44)	*ANOVA*	*Partial eta squared*	*Power*
Age (years)	43.2 ± 15.8 (22‒78)	47.6 ± 12.1 (28‒75)	45.9 ± 13.0 (26‒87)	F(2177) = 1.45; *p* = 0.236	0.016	0.308
Body mass (kg)	83.2 ± 12.8^a^ (63‒120)	76.7 ± 10.0 (61‒98)	75.2 ± 10.5 (56‒101)	F(2177) = 8.23; *p* < 0.001	0.090	0.959
Height (m)	175.8 ± 7.9 (160.0‒195.0)	175.0 ± 6.8 (160.0‒189.0)	176.2 ± 7.6 (161‒194)	F(2177) = 0.25; *p* = 0.777	0.003	0.089
BMI (kg/m^2^)	26.9 ± 24.0^a^ (17.6‒40.2)	24.8 ± 2.3 (21.0‒31.3)	23.9 ± 2.6 (16.9‒29.3)	F(2177) = 11.81; *p* < 0.001	0.127	0.994
Weekly moderate activity (min)	28.7 ± 37.8^b^ (0‒140)	136.8 ± 112.7^b^ (0‒480)	358.8 ± 522.8 (0‒1920)	F(2177) = 23.14; *p* < 0.001	0.208	1.000
Weekly vigorous activity (min)	8.8 ± 20.4^a^ (0‒90)	192.8 ± 64.4^b^ (0‒270)	544.5 ± 450.8 (0‒2520)	F(2177) = 85.4; *p* < 0.001	0.491	1.000

Data are presented as mean ± standard deviation (min‒max).

a *p* < 0.05 (different from Group 2 and 3).

b *p* < 0.05 (different from Group 2). BMI, Body Mass Index.

Comparing the results of the self-reported physical, mental, and emotional health status evaluated with the SF-36 questionnaire, significant differences were observed among the three groups. The Bonferroni post hoc test showed that Group 1 presented worse results than Groups 2 and 3 for physical functioning (mean difference = 13.5, 95 % IC 20.9 to 6.1, *p* < 0.001), physical role functioning (mean difference = 15.0, 95 % IC 27.1 to 3.0, *p* = 0.009), bodily pain (mean difference = 17.8, 95 % IC 26.1 to 9.5, *p* < 0.001), general health perception (mean difference = 15.7, 95 % IC 23.1 to 8.3, *p* < 0.001), vitality (mean difference = 18.8, 95 % IC 27.4 to 10.3, *p* < 0.001), social functioning (mean difference = 17.1, 95 % IC 26.7 to 7.5, *p* < 0.001), emotional role functioning (mean difference = 21.9, 95 % IC 37.7 to 6.2, *p* = 0.003), mental health (mean difference = 18.1, 95 % IC 24.7 to 11.5, *p* < 0.001), overall physical domain (mean difference = 15.5, 95 % IC 22.1 to 8.9, *p* < 0.001) and overall mental domain (mean difference = 19.1, 95 % IC 27.5 to 10.6, *p* < 0.001). Conversely, Groups 2 and 3 presented no significant difference in any of the domains: physical functioning (mean difference = 0.5, 95 % IC 9.1 to −8.0, *p* = 1.00), physical role functioning (mean difference = −0.7, 95 % IC 13.2 to −14.7, *p* = 1.00), bodily pain (mean difference = −5.2, 95 % IC 4.3 to −14.9, *p* = 0.566), general health perception (mean difference = 3.2, 95 % IC 11.8 to −5.3, *p* = 1.0), vitality (mean difference = −0.4, 95 % IC 9.5 to −10.4, *p* < 0.001), social functioning (mean difference = −4.9, 95 % IC 6.2 to −16.1, *p* = 0.860), emotional role functioning (mean difference = 4.1, 95 % IC 14.1 to −22.4, *p* = 1.00), mental health (mean difference = −3.1, 95 % IC 4.6 to −10.7, *p* = 1.00), overall physical domain (mean difference = −0.5, 95 % IC 7.1 to −8.2, *p* = 1.00) and overall mental domain (mean difference = 3.1, 95 % IC 12.9 to −6.6, *p* = 1.00) (Table 2).

**Table 2:** Short form health survey (SF-36).

	Group 1 (*n* = 95)	Group 2 (*n* = 41)	Group 3 (*n* = 44)	*ANOVA*	*Partial eta squared*	*Power*
Physical domain						
Physical functioning	83.6 ± 22.0^a^ (20‒100)	97.2 ± 3.7 (90‒100)	97.7 ± 4.1 (85‒100)	F(2177) = 16.0; *p* < 0.001	0.153	1.000
Physical role functioning	79.4 ± 33.0^a^ (0‒100)	94.5 ± 15.3 (25‒100)	93.7 ± 17.9 (25‒100)	F(2177) = 6.7; *p* = 0.001	0.071	0.915
Bodily pain	66.3 ± 19.8^a^ (31‒100)	84.1 ± 15.9 (31‒100)	78.9 ± 17.1 (41‒100)	F(2177) = 15.9; *p* < 0.001	0.153	1.000
General health perception	70.1 ± 18.9^a^ (5‒100)	85.7 ± 15.3 (35‒100)	89.0 ± 9.7 (67‒100)	F(2177) = 25.7; *p* < 0.001	0.225	1.000
Overall Physical domain	74.9 ± 18.2^a^ (30.7‒99.2)	90.4 ± 9.5 (59.5‒100)	89.5 ± 8.4 (59.5‒100)	F(2177) = 24.2; *p* < 0.001	0.215	1.000
Mental domain						
Vitality	58.1 ± 23.0^a^ (15‒95)	76.9 ± 13.6 (30‒100)	76.5 ± 11.8 (45‒100)	F(2177) = 21.5; *p* < 0.001	0.196	1.000
Social functioning	76.6 ± 25.8^a^ (25‒100)	93.7 ± 12.1 (50‒100)	88.7 ± 16.0 (50‒100)	F(2177) = 11.0; *p* < 0.001	0.111	0.901
Emotional role functioning	71.5 ± 41.8^a^ (0‒100)	93.5 ± 20.0 (0‒100)	89.4 ± 28.5 (0‒100)	F(2177) = 7.3; *p* = 0.001	0.077	0.936
Mental health	66.5 ± 16.8^a^ (28‒100)	84.7 ± 11.5 (48‒100)	81.6 ± 11.3 (44‒100)	F(2177) = 29.3; *p* < 0.001	0.250	1.000
Overall mental domain	68.1 ± 22.5^a^ (20.2‒95.5)	87.2 ± 11.4 (49.7‒100)	84.0 ± 13.9 (41‒100)	F(2177) = 19.8; *p* < 0.001	0.184	1.000

Data are presents as mean ± standard deviation (min‒max). ^a^*p* < 0.05 (different from Group 2 and 3).

**Fig. 1:**
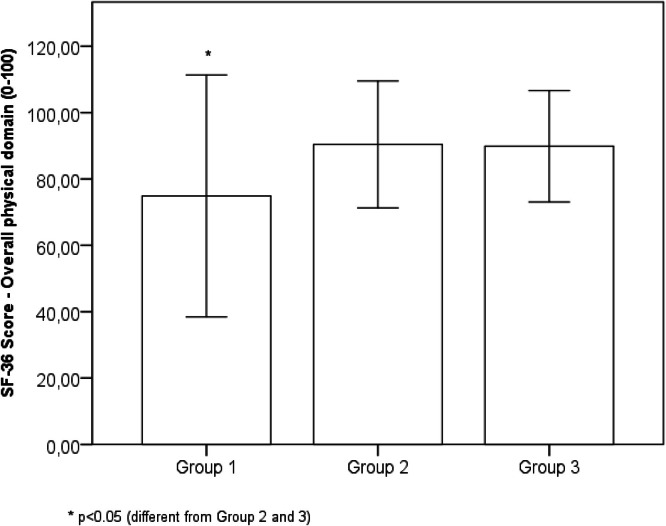
Score in overall physical domain obtained for Groups 1, 2 and 3. Data are presented as the mean ± standard deviation.

**Fig. 2:**
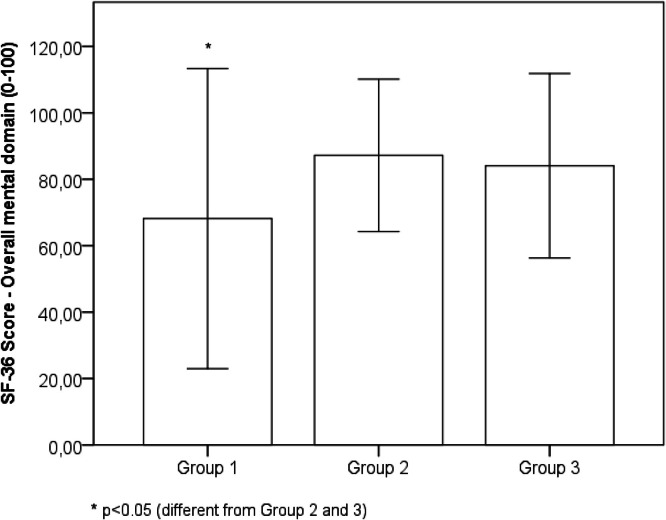
Score in overall mental domain obtained for Groups 1, 2 and 3. Data are presented as the mean ± standard deviation.

Likewise, the overall physical domain (Fig. 1) and mental domain (Fig. 2) showed significant improvement from Group 1 to Group 2; however, there was no significant difference between Groups 2 and 3.

## Discussion

In the last two decades, important evidence has emerged that physical activity should be considered as a medicine in the treatment and prevention of several noncommunicable diseases. For this reason, more recently, the WHO updated and increased the minimum recommendation for the weekly time dedicated to physical activity. However, it is unknown if an even greater time and/or intensity than the current weekly recommendation can produce even greater physical or mental benefits. Therefore, the aim of the present study was to compare the QoL, assessed by the SF-36, among individuals spending different time with physical activity during the week.

The main results of the study were: (i) Individuals who spent 150–600 min with moderate physical activity or 75–300 min with vigorous activity weekly presented better physical and mental health than those who did not reach these times with physical activity; and (ii) Individuals who spent > 600 min with moderate or > 300 min with vigorous physical activity did not present any additional physical or mental benefits. Therefore, the initial hypothesis was confirmed: that physical exercise is beneficial for QoL but that excessive exercise (more than twice the WHO recommendation) does not promote additional gains.

Physical inactivity is the fourth leading risk factor for global mortality^4^. Despite researchers' and health organizations' efforts, like the WHO, to disseminate knowledge about the health benefits of physical activity, participation levels among the entire population are decreasing, especially after the COVID-19 pandemic, which had a significant negative impact^16^^,^^17^.

The present results showed that the group of individuals who met or slightly exceeded the physical activity recommendation determined by the WHO present significantly better results in eight different domains associated with QoL (namely: physical functioning, physical role functioning, bodily pain, general health perception, vitality, social functioning, emotional role functioning and mental health) than those individuals who practice less physical activity than the WHO recommendation. These findings reinforce the importance of physical activity not only for physical health but also for mental health.

There are several complex interactions between physical activity and the neurobiological mechanisms underlying mental health. Among the proposed hypotheses are the increased production of neurotrophic factors, such as brain-derived neurotrophic factor (BDNF) or insulin-like growth factor-1 (IGF-1), during physical activity, which contribute to improving brain function^18^, particularly in domains related to cognition and memory^19^^,^^20^. Another relevant hypothesis relates to the anti-inflammatory and antioxidant effects of exercise. While chronic stress is known to increase systemic inflammation, which has been associated with depression and anxiety, regular physical activity appears to reduce this inflammation response^21^^,^^22^, thereby promoting mental health. Moreover, physical activity has been shown to increase the release of monoamines, such as serotonin and dopamine, which play a key role in mood regulation and have recognized antidepressant effects^23^. In addition, the role of endorphins and endocannabinoids has also been proposed. The endorphin hypothesis, originally formulated to explain the feeling of euphoria and pleasure following physical activity[18] was based on studies showing a strong association between plasma endorphin levels and mood improvements^24^. Although this theory was later challenged due to the inability of endorphins to cross the blood-brain barrier (BBB) and directly activate central opioid receptors^25^, more recently, findings suggest that exercise may enhance opioid receptor binding within the central nervous system, thereby inducing euphoria^26^. Finally, there is also the hypothesis related to the endocannabinoid system. Physical activity would cause an increase in blood concentrations of cannabinoids, such as anandamide, for example, which stimulates cannabinoid receptors, causing a reduction in anxiety and an improvement in well-being.

In contrast, participants who practice more than twice the WHO recommendation of physical activity do not present any additional benefits to QoL (neither physical nor mental). These results suggest that despite the importance of physical activity for QoL, there is no linear relationship between the two. It is possible that there is an optimal quantity of exercise above which no additional benefits can be observed, and the various possible neurobiological mechanisms mentioned above to explain the relationship between physical activity and mental health may have a ceiling effect. In Group 3 of the present study, participants practiced moderate physical activity for a mean of 6 h/week and vigorous physical activity for 9 h/week. It is possible that high exercise volumes or intensities are associated with a higher incidence of orthopedic injuries, which may mitigate the benefits of physical activity for QoL, as has been demonstrated previously^9^^,^^10^. Likewise, recent findings suggest that very high volume/intensity long-term exercises may be associated with potentially adverse cardiovascular manifestations, such as accelerated coronary artery calcification, exercise-induced cardiac biomarker release, myocardial fibrosis, and atrial fibrillation^27^, which may attenuate the health benefits of a physically active lifestyle^27^. There are also some previous findings associating very intense exercise with alterations in oxidative stress and immunological markers, which may also mitigate the health benefits of physical activity^28^^,^^29^.

Another possible negative effect of excessive intensity/volume of physical activity is a higher incidence of relative energy deficiency in sport (RED-S), which is a syndrome characterized by deleterious health and performance outcomes experienced by some athletes exposed to a very high intensity/volume of training and therefore high exercise energy expenditure without sufficient energy intake, resulting in low energy availability^30^. This condition is associated with several negative health effects, including, but not limited to, reduced sleep quality, mood disturbances, depressive symptoms, and subjectively reported reduced well-being, as well as detrimental effects in musculoskeletal health, reproductive function, immunity, glycogen synthesis, and cardiovascular and hematological health^31^, ^32^, ^33^, ^34^, ^35^.

## Practical application

Health professionals should encourage the population to practice physical activity to improve QoL, both in physical and mental aspects. However, they should know that physical activity of very high intensity/volume (more than twice the WHO recommendation) does not produce additional benefits for QoL.

## Strengths and limitations

A strength of the study is the large sample size, the use of validated questionnaires, and the wide range of physical activity among the participants. However, the study is not free from limitations, this is a cross-sectional study and therefore cannot be used to infer causality between physical activity and QoL because a temporal sequence cannot be established. Secondly, only male subjects took part in the study.

Therefore, the authors suggest that future longitudinal studies including female participants should be performed.

## Conclusion

The present findings confirm that adherence to WHO PA recommendations is associated with optimal QoL, both in physical and mental aspects. However, those who practice more than twice the minimum recommendation do not experience additional benefits. The authors advocate for the development of longitudinal studies, with a particular focus on the female population.

## Authors’ contributions

Conceptualization: R.A.C., M.S.A.; data curation: R.A.C.; formal analysis: R.A.C., M.S.A.; funding acquisition: M.S.A.; investigation: R.A.C.; methodology: R.A.C.; project administration: M.S.A.; supervision: M.A.S.; validation: C.A.B.L., R.L.V.; visualization: C.A.B.L., R.L.V.; roles/writing-original draft: R.A.C., M.S.A., K.W., B.K.; and writing-review & editing: R.A.C., M.S.A., C.A.B.L., R.L.V., K.W., B.K.

## Declaration of competing interest

The authors declare no conflicts of interest.
